# Thoracic Spine Pain in High School Adolescents: A One-Year Longitudinal Study

**DOI:** 10.3390/healthcare11020196

**Published:** 2023-01-09

**Authors:** Alberto De Vitta, Matias Noll, Manuel Monfort-Pañego, Vicente Miñana-Signes, Nicoly Machado Maciel

**Affiliations:** 1 Departament of Physical Therapy, Centro Universitário das Faculdades Integradas de Ourinhos, Rodovia BR 153, Km 338 + 420 m, Água do Cateto, Ourinhos 19909-100, SP, Brazil; 2 Instituto Federal Goiano, Rodovia GO 154, Km 03, s/n, Ceres 76300-000, GO, Brazil; 3 Faculdade de Educação Física e Dança, Universidade Federal de Goiás, Goiânia 74001-970, GO, Brazil; 4 Facultat de Magisteri, Universitat de València, Av. dels Tarongers, 4, 46022 Valencia, Spain; 5 Department of Health Sciences, University of São Paulo, Ribeirão Preto 14049-900, SP, Brazil

**Keywords:** adolescent, epidemiology, thoracic spine pain, musculoskeletal disorders, risk factors

## Abstract

Thoracic spine pain (TSP) is a common condition in the general adult population, with a similar prevalence in children and adolescents. An in-depth understanding of risk factors can assist in the identification of potential targets for effective prevention strategies. This study aimed to determine the incidence of TSP and ongoing TSP and identify its predictors in high school students. This longitudinal study was conducted in 2017 (baseline-T1), and follow-up surveys were completed in 2018 (T2). The variable “thoracic spine pain” was observed using the Nordic questionnaire, and associated variables were observed through the Baecke questionnaire and the Strengths and Difficulties Questionnaire. Statistical association methods were used for bivariate and multivariate logistic regression analysis. Among the participants, the one-year prevalence (ongoing TSP) was 38.4%, and the one-year incidence (new TSP) was 10.1%. Significant risk factors for ongoing TSP were adolescent females (RR = 2.14), in the age group of 15 to 18 years (RR = 1.41), clinical mental health problems (RR = 3.07), borderline mental health problems (RR = 2.02), mental health problems, sitting while using a tablet (RR = 1.93), distance of the eye from cell phone screen of or more than 20 cm (RR = 1.69), distance of the eye from the PC screen of or more than 30 cm (RR = 1.53), cell phone mobile use duration of or more than 3 h (RR = 1.60), tablet use time of or more than 3 h (RR = 2.08), and semi-lying prone position while using the cell phone (RR= 1.47), and these were also significant predictors of TSP episodes. Significant risk factors for new TSP were adolescent female sex (RR = 1.88), level, clinical mental health problems (RR = 4.26), borderline mental health problems (RR = 2.07), semi-lying prone position while using cell phone (RR = 1.71) or tablet (RR = 2.31), and mobile phone use duration equal to or greater than 3 h (RR = 1.72). We conclude that there is a high prevalence of TSP in high school students, which is associated with the female sex, mental health problems, and use of electronic devices for an inappropriate duration in an improper position.

## 1. Introduction

Thoracic spine pain (TSP) is common in different age groups of the general population worldwide [1], with the prevalence in adults ranging from 15–35% and in children and adolescents from 13–35% [2].

Physical, physiological, psychological, and behavioral risk factors or a combination of these are associated with TSP, according to various investigations [3,4,5,6]. There is also strong evidence of the effects of physical activity, sedentary behavior, and mental health on spinal health. All these factors have been considered as critical by the World Health Organization (WHO) in its last global guidelines evidence review [7].

The technological development in advanced societies is imposing an intense relationship between the human body and electronic devices. Therefore, it is imperative to study the consequences of this interaction in the musculoskeletal system, as little international data [8] are available regarding its relationship with TSP. To our knowledge, no Latin American studies have evaluated the relationship between electronic devices with TSP. In addition, there are consistent data on the association of electronic device use with lower-back and neck pain. A natural follow-up to explore the association of electronic device use with TSP should be done once we know the interference of one spinal region with the other due to their connections. The most recent review on TSP [9] showed that poor mental health and age transition from early to late adolescence were two of the most important risk factors related to TSP.

Information on risk factors for TSP in secondary school students is relevant for the following reasons: children and adolescents with back pain use health care services more often, they are more inactive, they show low academic achievement, and they exhibit more psychosocial problems [9,10,11]. Additionally, few studies have been conducted on TSP compared with that on low-back and neck pain. A systematic TSP review identified only two prospective studies regarding prognostic factors [12]. In addition to these two previous reasons, studies of this nature contribute to the design and implementation of preventive strategies, primarily on modifiable factors.

Considering theoretical reference, the aim of this study was to determine the incidence of TSP and ongoing TSP and identify the predictors (sociodemographic variables, electronic devices, level of physical activity, and mental health) of TSP in high school students.

## 2. Materials and Methods

### 2.1. Design and Population

This longitudinal study used data from baseline (2017) and follow-up surveys (2018) conducted on 14- to 18-year-old male and female students attending their first- and second-class years of high school during the day in the urban area of Bauru, São Paulo, Brazil. This study was approved by the Research Ethics Committee of the University of Sagrado Coração (no. 1972579) and is part of “Back pain and associated factors in students of high school: a longitudinal study”, which was used to develop studies on the frequency and incidence of low-back pain and TSP.

### 2.2. Sample Calculation

This study was based on the data collected by the project “Incidence and factors associated with low back pain in adolescents: A prospective study” (Financed by Fapesp, Process: 2016/182837), with 14- to 18-year-olds of both sexes attending the first and second years of high school in the morning in the urban area of Bauru, SP, Brazil.

The previous study involved one sample (*n* = 1366) per conglomerate in two stages, where the primary sampling units (PSU) are the schools and secondary sampling units (SSU) are the classes concerning the three years of secondary education of the selected schools. The sample of school children was formed, therefore, by all the students of SSU classes selected in the sample of schools PSU. The criteria adopted for the exclusion of some schools randomly selected for the study were: age below 14 years and above 18 years; non-submission of informed consent form signed by parents/guardians; and refusal against participation [13,14].

Considering the inclusion and exclusion criteria, the participants in this study were 1628 students who answered the questionnaire in the period of March to June 2017.

### 2.3. Inclusion and Exclusion Criteria

Students who participated in our baseline and follow-up studies in 2017 and 2018 and answered the questionnaire alone, were 14 to 18 years of age, and whose parents provided informed consent were included in this longitudinal study. Students were excluded from the study if they no longer attended school during the day (and instead attended at night), dropped out of school completely, or were not present when we visited the school three times to complete the study [14].

### 2.4. Variable Description

Outcome

TSP was assessed by the validated standardized Brazilian version of the Nordic questionnaire [15]. In the present study, TSP pain was defined as non-specific pain experienced in the column spinal extending from the cervicothoracic joint (C7–T1) to the thoracolumbar junction (T12–L1) and the corresponding posterior aspect of the trunk and that could occasionally cause radiating pain in the anterior chest wall unrelated to trauma, tumors, or other diseases [12].

During the baseline interview in 2017, students answered the following question: “In the last 12 months (until 1 year ago), did you have pain in some of these regions of the thoracic spine (in the areas shown in the body chart)?” During the follow-up interview in 2018, students answered the same question, but the timeframe of the question was changed to specifically cover the period since the baseline assessment in 2017. For example, if a student was first assessed in March 2017 (baseline interview), then the student was interviewed again in March 2018 (follow-up interview) regarding the occurrence of any episode of TSP between March 2017 and March 2018.

In addition to the verbal questionnaire, an image of the spinal region with different colors was presented to help the interviewees in specification and identification of spinal areas (lumbar, thoracic, and cervical) [16]. The students could choose more than one answer; however, during the study, only thoracic spinal data were used.

### 2.5. Explanatory Variables

Variables evaluated with the same questions in 2017 and 2018 were considered independent during this study according to the conceptual theoretical hierarchical model [17,18]. To construct this model, the variables were organized to consider their temporal and causal relationships with occurrence. Considering the factors associated with pain in the literature, the present study organized these determinants into four levels (level 1: sex, age, race, and socioeconomic level; level 2: health variable (mental health); level 3: variables related to the use of electronic devices; level 4: physical activity).

For sociodemographic purposes, we collected data on sex, age, and skin color (classification as defined by the Brazilian Institute of Geography and Statistics).

Questions were asked regarding the use of electronic devices [19] (Table 1). Questions about posture (such as “What posture do you have while using mobile phones?” and “What posture do you have while using the tablet?”) were asked, and each posture was transformed into a variable. To answer the above questions, the participants were categorized into a group of individuals who used the equipment in the posture in question (yes) and a group of individuals who did not (no). A pilot study involving 42 high school students who did not participate in the study was conducted to measure reproducibility, and all responses showed good values (κ range, 0.66–0.88) [20].

The Baecke questionnaire validated in Brazil (Baecke Habitual Physical Activity Questionnaire) was used for measuring the level of physical activity of the students. The scores obtained were subdivided into quartiles according to the individual total score, and thus, they were classified as sedentary (first quartile), moderately active (second and third quartile), and active (fourth quartile) [21].

The Strengths and Difficulties Questionnaire validated in Brazil was used to assess adolescent mental health [22]. The questionnaire contains 25 items grouped into five scales (hyperactivity scale, emotional symptoms, behavior problems, relationship problems, and prosocial behavior) containing five items each. Of these 25 items, 10 and 14 pertain to skills and difficulties, respectively, while one is considered neutral. Each item can be answered as “false”, “more or less true”, or “true”. The score for each scale was obtained by summing scores of the five items, thus generating a score that varied from 0 to 10. Scores for the hyperactivity scales, emotional symptoms, and behavioral and peer relationship problems were summed to generate a total score for difficulties (range, 0–40). We considered a total score of 20 or more as “abnormal” (clinical), indicating that there are great difficulties related to what is being evaluated, requiring specialized intervention; a total score between 16 and 19 was considered “limited”, indicating that the child or adolescent already has some difficulty that, if not properly cared for, may deteriorate and impair their development; and a total score of 15 or less was considered “normal” [22,23].

### 2.6. Data Collection Procedure

After parental/guardian consent was obtained, baseline data were collected from March to June 2017 by trained assessors who were undergraduate and graduate students. After meeting with the heads of all schools and receiving their authorization, the researchers distributed questionnaires to the classrooms for initial evaluation (T1). Data were collected by trained raters in each classroom, where they explained the objectives of the study, that student participation was voluntary, and that the students’ participation would be confidential. Students were then instructed on how to complete and informed that responses were individual. No contact between them was allowed, and the average time to complete was 60 min.

Follow-up (T2) data were collected one year later between March and June 2018 using the same procedures as those used during the baseline assessment regardless of the class year of the student during 2018.

At each school, three extra visits were conducted to collect data from students who were absent from classes related to previous collections, and three telephone contacts were attempted for those who left the school, changed cities, or changed schools. Students who were not present during the three visits or who could not be reached after three telephone contact attempts were considered lost. Students who refused to answer the questionnaire were considered as refusals.

### 2.7. Data Analysis

The data were analyzed using Statistical Package for the Social Sciences version 26.0 (SPSS, Chicago, IL, USA), entered by an independent researcher. Further, 10% of the questionnaires were randomly chosen to check the accuracy of data entry, and another set of 5% of the questionnaires were randomly chosen, and no errors were found.

Descriptive analysis was performed, in which prevalence and confidence intervals were calculated. Bivariate and multivariate logistic regression analyses of TSP (dependent variable) and all independent variables were used to verify the association between them. The significance level, estimated risk ratio (RR), and 95% confidence intervals (CIs) were determined.

Students who had never reported TSP were compared with those who had reported TSP during the second evaluation (T2) (no TSP during either T1 or T2 vs. new TSP). Additionally, students who reported that they had never experienced TSP were compared with those who reported TSP during T1 and T2 (no TSP during either T1 or T2 vs. TSP during both T1 and T2) [24].

The hierarchical conceptual theoretical model was used for the bivariate and logistic regression analyses. The variables were organized into four levels according to temporal and causal relationships for TSP. The first level was adjusted for using all variables belonging to that level. The second level was adjusted for using the variables of the first level with values of *p* < 0.10 after adjustment and the variables belonging to the second level. The third level was adjusted for using the variables of the first and second levels with values of *p* < 0.10 after adjustment and the variables belonging to the third level. Finally, the fourth level was controlled using the variables of the first three levels. To select the variables that would remain in the regression model, the backward selection process was used, and the final model included all variables with values of *p* < 0.05 [17,18].

## 3. Results

**Figure 1:**
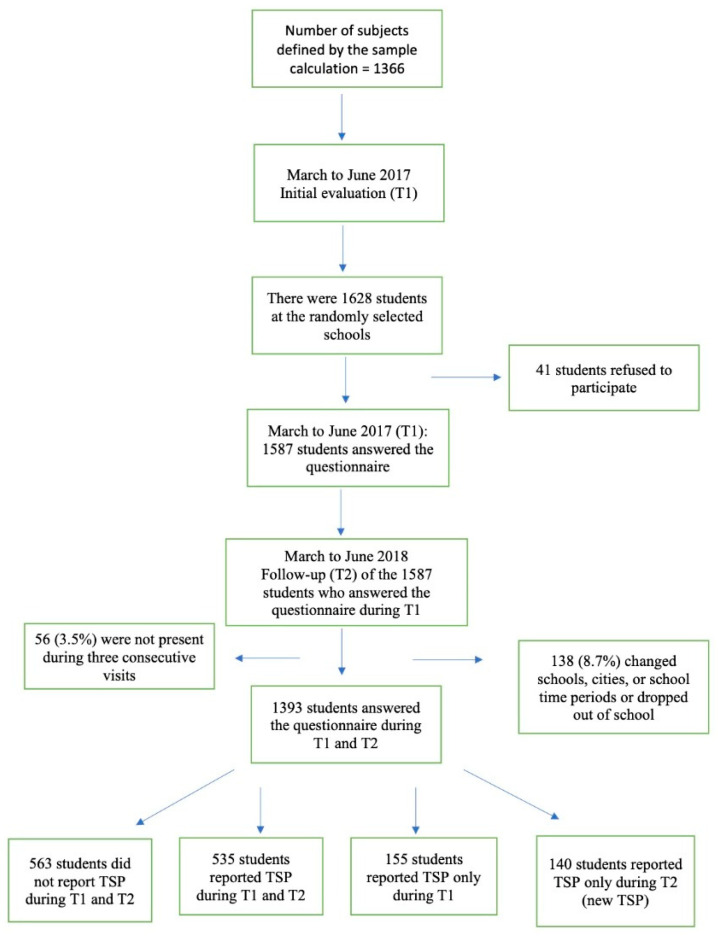
Flow chart of study participants (TSP, thoracic spine pain).

In 2017 (T1), 1628 students were randomly selected; 41 (2.5%) of these students refused to answer the questionnaire. In 2018 (T2), out of the 1587 students who answered the questionnaire during T1, 138 (8.7%) changed schools, cities, or school time periods or dropped out of school, and 56 (3.5%) were not present during three consecutive visits. Therefore, the results of 1393 adolescents interviewed during T1 were analyzed during T2 (Figure 1).

Of all participants, the one-year prevalence was 38.4%, meaning that adolescents reported TSP during T1 and T2 (ongoing TSP). In addition, 155 adolescents (11.2%) who reported TSP at T1 did not report TSP at T2. The one-year incidence was 10.1%; i.e., they did not report TSP during T1, and they were referred as new cases of TSP (new TSP). Table 2 shows the socio-demographic characteristics and use of electronic equipment at both times.

After performing the multivariate logistic regression comparing no TSP during either T1 or T2 (score = 0) with TSP during both T1 and T2 (score = 1), the following variables remained risk factors for TSP (ongoing TSP): adolescent females (RR = 2.14; CI:1.26–2.81) and in the age group of 15 to 18 years (RR = 1.41; CI:1.01–2.00) were the only variables that remained in the model and were used as a potential covariate for the adjusted analysis in the second level. In the analysis of the second level, clinical (RR = 3.07; CI:2.17–4.34) and borderline (RR = 2.02; CI:1.48–2.76) mental health problems remained significant predictors of TSP events and were used as a potential covariate along with sex for the adjusted analysis in the third level. The third level showed that sitting while using a tablet (RR = 1.93; CI:1.14–1.90), distance of the eye from cell phone screen of or more than 20 cm (RR = 1.69; CI:1.17–2.50), distance of the eye from the PC screen of or more than 30 cm (RR = 1.53; CI:1R14–2.04), cell phone mobile use duration of or more than 3 h (RR = 1.60; CI:1.21–2.13), tablet use time of or more than 3 h (RR = 2.08; CI:1.01–4.34), and semi-lying prone position while using the cell phone (RR= 1.47 (1.14–1.90)) were significant predictors of TSP episodes (Table 3).

After performing multivariate logistic regression comparing no TSP during either T1 or T2 (score = 0) with TSP only during T2 (score = 1), the following variables remained risk factors for TSP: adolescent female sex (RR = 1.88; CI:1.26–2.81) was the only variable that remained in the model and was used as a potential covariate for the adjusted analysis in the second level. In the analysis of the second level, clinical (RR = 4.26; CI:2.63–6.89) and borderline (RR = 2.07; CI:1.27–3.36) mental health problems remained significant predictors of new TSP events, and they were used as a potential covariate along with sex for the adjusted analysis in the third level. The third level showed that semi-lying prone position while using cell phone (RR = 1.71; CI:1.17–2.52) and tablet (RR = 2.31; CI:1.02–5.24) and mobile phone use duration of or more than 3 h (RR = 1.72; CI:1.08–2.74) were significant predictors of new TSP episodes (Table 4).

## 4. Discussion

This study aimed to determine the incidence of TSP (new TSP) and ongoing TSP in high school students and to identify its predictors (sociodemographic variables, electronic device use, physical activity, and mental health). This is the first study in Brazil to longitudinally examine the role of factors in the onset of new and ongoing TSP in young people.

In the present study, TSP was significantly associated with adolescent females aged 15 to 18 years over those aged 14 years or younger. Clinical and borderline mental health problems, semi-lying prone position while using cell phone, sitting while using a tablet, distance of the eye from the cell phone screen of or more than 20 cm, distance of the eye from the PC screen of or more than 20 cm, mobile phone use duration of or more than 3 h, and tablet use duration of or more than 3 h were significant predictors of ongoing TSP episodes. The main predictors of new episodes of TSP were adolescent female sex, clinical and borderline mental health problems, semi-lying prone position while using cell phone and tablet, and mobile use duration of or more than 3 h.

During this study, 38.4% of the adolescents reported ongoing TSP, and 10.1% reported new episodes of TSP. Epidemiological data, especially on TSP incidence in the general population, are extremely limited. Some data on TSP prevalence have been reported in other countries such as Australia (20.0%) [25], Portugal (13.2%) [8], southern Denmark (36%) [26], and Brazil (51.5%) [13].

Different explanations for this have been suggested in the literature. First, it is possible that the TSP experienced by adolescents is so benign, and their natural history is so favorable that memory of the episode fades or, according to Savedra et al. [27], pain vocabulary changes with cognitive development. Goodman and McGrath [28] recommend avoiding long timescales because they can cause recall bias, with respondents forgetting the back problems they experienced during previous years. Of the variables investigated, some showed a relationship with persistent TSP or the occurrence of new TSP episodes in adolescents in the multivariate model. These possibilities are consistent with the literature regarding this subject [24].

Sex was associated with ongoing TSP (no TSP during either T1 or T2 vs. TSP during both T1 and T2) and new TSP (TSP during T1 or T2 (score = 0) vs. TSP only during T2 (score = 1)). Cross-sectional studies with adolescents showed results similar to those of the present investigation [8,10,25,29]. There are numerous possible explanations for the association between TSP and sex. First, it is believed that boys have a higher pain threshold than girls during puberty [30]. Second, hormonal changes caused by female puberty occurring earlier than male puberty may interfere with a girls’ perception of pain [31]. Finally, from a social perspective, it is usually more acceptable for girls to express their symptoms and feelings [31].

In the current study, mental health was associated with ongoing TSP and new TSP. Transversal studies have demonstrated an association between TSP and mental health [3,32,33], which may explain the results of this study. Based on the fear-avoidance model [34], Harvtvigsen et al. [35] explained that pain cognition could play an important role in the development of disability. Self-efficacy, psychological distress, and fear are intermediate factors between back pain and disability [34,36].

Factors present in the school environment, such as pressure, economic difficulties, and relationship problems, can contribute to the onset of emotional symptoms. These psychosomatic symptoms can manifest in different ways, such as headaches, abdominal pain, and musculoskeletal problems, including pain in the spine [3,32,37].

Despite a rise in use of technologies, the effect of body interaction with screens and electronic devices has not been an important research topic in the last ten years [38]. In this sense, our study analyzed a significant number of modifiable factors related to body posture and use of electronic devices, that influence back health.

As in previous studies [19,32,39], our study showed that students exceeded the maximum time of 2 h/day in front of a screen recommended by the World Health Organization guidelines [7] and that the cell phone hours/day is strongly associated with ongoing and new TSP; however, in the case of tablet hours/day, the association occurs only with ongoing TSP. It is possible that this risk factor is mediated by other confounding variables [40]. It should be noted that, in our study, the confounding factor variables were adjusted according to their behavior at different levels.

Body postures using electronic devices are another factor contributing to musculoskeletal complaints [38]. Our results showed that the semi-lying prone posture while using a cell phone was strongly associated with ongoing and new TSP cases. During the analysis of new TSP cases, semi-lying prone position while using tablets was included in the multivariate model; however, it was associated with ongoing TSP only. These results can be explained by the degree of spinal flexion during semi-lying prone posture. Sustained spine flexion is one of the key factors that explains the prevalence of back pain [40].

The distance of the eye from the screen is another factor related to pain. Our results showed that the distances of the eye from the cell phone and PC screen were associated with ongoing TSP. Shan et al. [19] found similar results; long-term use and short eye-to-screen distance were also related to back pain because of higher spine flexion.

Incorrect use of equipment and maintaining improper postures for long periods, such as flexion of the cervical and thoracic spine, increases stress on the thoracic and lumbar regions since all the muscles and dorsal structures of the spine work together to provide stability and movement to the trunk and limbs [41]. These inadequate postures during the use of electronic equipment promote an increase in the compression of the intervertebral discs, biomechanical alterations such as shoulder protrusion, and scapular dysfunction during the movement of the upper limbs, contributing to an increase in thoracic kyphosis and, consequently, a higher risk of TSP [42,43,44,45].

It is important to highlight the limitations of this study. Memory bias may be present in the responses presented, as the data were collected based on self-reported responses [46]. There are confounding factors that could not be controlled, such as specific types of electronic devices used, other technologies used, and previous injuries. Finally, the students evaluated were selected from public schools, which limits the generalizability of these data to students from private schools.

It is important to note that validated questionnaires were used in this study. Moreover, TSP has been studied to a lesser extent than lower back or neck pain in the high school student population. This is one of the first longitudinal studies on TSP conducted on a large population of high school students in Brazil. However, future studies should address the problem of back health in all regions, as the spine is an integrated and interrelated system that requires an overall analysis of its functionality [47]. Another important aspect is that the incidence of TSP was relatively high in this population; however, it was associated with modifiable risk factors, such as positioning while using computers, cell phones, and tablets, as well as the duration of use of these electronics. Therefore, the development of preventive interventions in the school environment could be promising for controlling such risk factors [48,49].

## 5. Conclusions

The present longitudinal study of adolescent students in Brazil identified a high prevalence and incidence of TSP in high school students. Moreover, the TSP is associated with the female sex, mental health problems, and body posture while using cell phones, tablets, and PCs as well as with the duration of use of cell phone and tablet. These variables are mostly modifiable risk factors and amenable to early intervention.

## Figures and Tables

**Table 1:** Questions and answers about electronic equipment use.

**TV habits**	
During a normal school week, do you watch TV?	Yes/no
How many times per week do you watch TV?	1 or 2 times, 3 or 4 times, 5 times, >5 times
How many hours per day do you watch TV?	Less than 1 h, 2 h, 3 h, 4 h, 5 h, >5 h
**Computer use**	
Do you use a computer?	Yes/no
What type of computer do you use?	Desktop/laptop
What is the height of your PC screen?	Eyes above the midpoint of the screen, approximately in the middle point of the screen, below the midpoint of the screen
How many times per week do you use a computer?	1 or 2 times, 3 or 4 times, 5 times, >5 times
How many hours per day do you use a computer?	Less than 1 h, 2 h, 3 h, 4 h, 5 h, >5 h
What is the eye-to-screen distance while using your computer?	<20 cm, 20–25 cm, 25–30 cm, >30 cm
**Cell phone use**	
Do you use a cell phone?	Yes/no
What posture do you have while using the mobile phone?	Standing, sitting, lying down, semi-lying down
Average daily time spent using the cell phone?	<1 h, 2–3 h, 3–4 h, >4 h
What is the eye-to-screen distance during the use of the cell phone?	<10 cm, 10–15 cm, 15–20 cm, >20 cm
**Tablet use**	
Do you use a tablet?	Yes/no
What posture do you have while using the tablet?	Standing, sitting, lying down, semi-lying down
Average daily time spent using the tablet?	<1 h, 2–3 h, 3–4 h, >4 h
What is the eye-to-screen distance while using the tablet?	<10 cm, 10–15 cm, 15–20 cm, >20 cm

PC, personal computer; TV, television.

**Table 2:** Descriptive statistics of the study groups at baseline and at one-year follow-up.

Factors	Baseline (T1)		Follow-Up (T2)	
	*n*	%	*n*	%
Age				
14 years or younger	216	15.1	216	15.1
15–18 years	1.177	84.5	1177	84.5
Sex				
Male	693	49.7	693	49.7
Female	700	50.3	700	50.3
Skin color				
White	690	49.5	690	49.5
Black	130	9.3	130	9.3
Brown	530	38.0	530	38.0
Yellow	43	3.1	43	3.1
Physical activity				
Active	369	26.5	357	25.6
Moderately active	646	46.3	684	49.1
Sedentary	378	27.2	352	25.3
Mental health				
Normal	800	57.5	763	54.8
Borderline	301	21.6	324	23.2
Clinical	292	20.9	306	22.0
Watch TV				
No	163	11.7	185	13.3
Yes	1230	88.3	1208	86.7
TV use/week				
≤2 times	321	26.1	372	30.7
≥3 times	909	73.9	836	69.3
TV hours/day				
≤2 h	645	52.4	682	56.5
≥3 h	585	47.6	526	43.5
Use PC				
No	264	19.0	354	25.4
Yes	1129	81.0	1039	74.6
Computer type				
Desktop	529	46.8	501	48.2
Laptop	600	53.2	538	51.8
PC screen height				
Eyes aligned with the top of the screen	291	25.7	268	25.7
Eyes below the top of the screen	838	74.3	771	74.3
Distance from the eye to the PC screen				
≤30 cm	788	69.7	710	68.3
≥30 cm	341	30.3	329	31.7
PC use/week				
≤2 times	418	37.1	565	54.3
≥3 times	711	69.9	474	45.6
PC hours/day				
≤2 h	512	45.3	471	45.3
≥3 h	671	59.4	568	54.6
Cell phone use				
No	34	2.4	44	3.1
Yes	1359	97.6	1349	96.9
Posture during cell phone use *				
Standing				
No	884	65.1	773	57.3
Yes	475	34.9	576	42.7
Sitting				
No	642	47.2	528	39.1
Yes	717	52.8	821	60.9
Lying prone				
No	563	41.4	464	34.4
Yes	796	58.6	885	65.6
Semi-lying				
No	898	66.0	802	59.4
Yes	461	34.0	547	40.6
Cell phone hours/day				
≤2 h	301	22.1	305	22.6
≥3 h	1058	77.9	1044	77.4
Distance from the eye to the cell phone screen				
≤20 cm	1209	88.9	1169	86.6
≥20 cm	150	11.1	180	13.4
Tablet use				
No	1120	80.4	1118	80.2
Yes	273	19.6	205	14.8
Posture during tablet use *				
Standing				
No	216	79.1	140	68.2
Yes	57	20.9	65	31.8
Sitting				
No	119	43.5	72	35.1
Yes	154	56.5	133	64.9
Lying prone				
No	148	54.2	98	47.8
Yes	125	45.8	107	52.2
Semi-lying				
No	210	76.9	139	67.8
Yes	66	24.1	66	32.2
Tablet hours/day				
≤2 h	208	76.1	161	78.5
≥3 h	65	23.9	44	21.6
Distance from the eye to the tablet screen				
≤20 cm	221	80.9	174	84.8
≥20 cm	52	19.1	31	15.2

* Participants could choose more than one answer. PC, personal computer; TV, television.

**Table 3:** Multivariate logistic regression comparing no TSP during either T1 or T2 (score = 0) with TSP during both T1 and T2 (score = 1).

Factor	Ongoing TSP	
	*p*-Value	RR (95% CI) *
Sex		
Male	0.0001	1.00
Female		2.14 (1.64–2.77)
Age		
14 years or younger	0.04	1.00
15–18 years		1.41 (1.01–2.00)
Cell phone hours/day		
≤2 h	0.001	1.00
≥3 h		1.60 (1.21–2.13)
Tablet hours/day		
≤2 h	0.04	1.00
≥3 h		2.08 (1.01–4.34)
Sitting while using the tablet		
No	0.04	1.00
Yes		1.93 (1.24–1.93)
Distance of the eye from the cell phone screen		
≤20 cm	0.005	1.00
≥20 cm		1.69 (1.17–2.50)
Distance of the eye from the PC screen		
≤30 cm	0.004	1.00
≥30 cm		1.53 (1.14–2.04)
Semi-lying prone position while using the cell phone		
No	0.003	1.00
Yes		1.47 (1.14–1.90)
Mental health		
Normal		1.00
Borderline	0.001	2.02 (1.48–2.76)
Clinical	0.0001	3.07 (2.17–4.34)

CI, confidence interval; RR, risk ratio; PC, personal computer; T1, initial evaluation; T2, follow-up; TV, television. * Final regression models all variables with values of *p* < 0.05: first level (sex): adjusted between them; second level (mental health): adjusted between them and for the first level; third level (electronic devices): adjusted between them and for the first- and second-level variables.

**Table 4:** Multivariate logistic regression comparing no TSP during either T1 or T2 (score = 0) with TSP only at T2 (score = 1).

Factor	Incidence of New TSP	
	*p*-Value	RR (95% CI) *
Sex		
Male	0.002	1.00
Female		1.88 (1.26–2.81)
Cell phone hours/day		
≤2 h	0.02	1.00
≥3 h		1.72 (1.08–2.74)
Semi-lying prone position while using cell phone		
No	0.005	1.00
Yes		1.71 (1.17–2.52)
Semi-lying prone position while using tablet		
No	0.04	1.00
Yes		2.31 (1.02–5.24)
Mental health		
Normal		1.00
Borderline	0.003	2.07 (1.27–3.36)
Clinical	0.0001	4.26 (2.63–6.89)

CI, confidence interval; RR, risk ratio; PC, personal computer; T1, initial evaluation; T2, follow-up. * Final regression models, all variables with values of *p* < 0.05: first level (sex): adjusted between them; second level (mental health): adjusted between them and for the first level; third level (electronic devices): adjusted between them and for the first- and second-level variables.

## Data Availability

Data available in a publicly accessible repository: FigShare.

## References

[1] Roberts N.L.S., Mountjoy-Venning W.C., Anjomshoa M., Banoub J.A.M., Yasin Y.J. (2019). GBD 2017 Disease and Injury Incidence and Prevalence Collaborators (2018). Global, regional, and national incidence, prevalence, and years lived with disability for 354 diseases and injuries for 195 countries and territories, 1990–2017: A systematic analysis for the Global Burden of Disease Study (vol 392, pg 1789, 2018). Lancet.

[2] Kjaer P., Wedderkopp N., Korsholm L., Leboeuf-Yde C. (2011). Prevalence and tracking of back pain from childhood to adolescence. BMC Musculoskelet. Disord..

[3] Batley S., Aartun E., Boyle E., Hartvigsen J., Stern P.J., Hestbæk L. (2019). The association between psychological and social factors and spinal pain in adolescents. Eur. J. Pediatr..

[4] García-Hermoso A., Ramírez-Campillo R., Izquierdo M. (2019). Is Muscular Fitness Associated with Future Health Benefits in Children and Adolescents? A Systematic Review and Meta-Analysis of Longitudinal Studies. Sports Med..

[5] Noll M., Wedderkopp N., Mendonça C.R., Kjaer P. (2020). Motor performance and back pain in children and adolescents: A sys-tematic review and meta-analysis protocol. Syst. Rev..

[6] Montgomery L.R.C., Kamper S.J., Hartvigsen J., French S.D., Hestbaek L., Troelsen J., Swain M.S. (2022). Exceeding 2-h sedentary time per day is not associated with moderate to severe spinal pain in 11- to 13-year-olds: A cross-sectional analysis. Eur. J. Pediatr..

[7] Bull F.C., Al-Ansari S.S., Biddle S., Borodulin K., Buman M.P., Cardon G., Carty C., Chaput J.-P., Chastin S., Chou R. (2020). World Health Organization 2020 guidelines on physical activity and sedentary behaviour. Br. J. Sports Med..

[8] Silva A.G., Sa-Couto P., Queirós A., Neto M., Rocha N.P. (2017). Pain, pain intensity and pain disability in high school students are differently associated with physical activity, screening hours and sleep. BMC Musculoskelet. Disord..

[9] Wirth B., Potthoff T., Rosser S., Humphreys B.K., De Bruin E.D. (2018). Physical risk factors for adolescent neck and mid back pain: A systematic review. Chiropr. Man. Ther..

[10] Heneghan N.R., Rushton A. (2016). Understanding why the thoracic region is the ‘Cinderella’ region of the spine. Man. Ther..

[11] Joergensen A.C., Strandberg-Larsen K., Andersen P.K., Hestbaek L., Andersen A.-M.N. (2021). Spinal pain in pre-adolescence and the relation with screen time and physical activity behavior. BMC Musculoskelet. Disord..

[12] Johansson M., Jensen Stochkendahl M., Hartvigsen J., Boyle E., Cassidy J. (2017). Incidence and prognosis of mid-back pain in the general population: A systematic review. Eur. J. Pain.

[13] Zar J.H. (2010). Biostatistical Analysis.

[14] de Vitta A., Bento T.P.F., Cornelio G.P., Perrucini P.D.D.O., Felippe L.A., de Conti M.H.S. (2021). Incidence and factors associated with low back pain in adolescents: A prospective study. Braz. J. Phys. Ther..

[15] Barros E.N.C., Alexandre N.M.C. (2003). Cross-cultural adaptation of the nordic musculoskeletal questionnaire. Int. Nurs. Rev..

[16] Hestbaek L., Iachine I.A., Leboeuf-Yde C., Kyvik K.O., Manniche C. (2004). Heredity of low back pain in a young population: A classical twin study. Twin Res. Hum. Genet..

[17] Ferreira G.D., Silva M.C., Rombaldi A.J., Wrege E.D., Siqueira F.V., Hallal P.C. (2011). Prevalence and associated factors of back pain in adults from southern Brazil: A population-based study. Braz. J. Phys. Ther..

[18] Victora C.G., Huttly S.R., Fuchs S.C., Olinto M.T. (1997). The role of conceptual frameworks in epidemiological analysis: A hierarchical approach. Int. J. Epidemiol..

[19] Shan Z., Deng G., Li J., Li Y., Zhang Y., Zhao Q. (2013). Correlational Analysis of neck/shoulder Pain and Low Back Pain with the Use of Digital Products, Physical Activity and Psychological Status among Adolescents in Shanghai. PLoS ONE.

[20] Chinapaw M.J., Mokkink L.B., van Poppel M.N., van Mechelen W., Terwee C.B. (2010). Physical activity questionnaires for youth: A systematic review of measurement properties. Sports Med..

[21] Guedes D.P., Lopes C.C., Guedes J.E.R.P., Stanganelli L.C. (2006). Reproducibility and validity of the Baecke questionnaire for assessing of the habitual physical activity in adolescents. Rev. Port. Cien. Desp..

[22] Fleitlich-Bilyk B., Goodman R. (2004). Prevalence of Child and Adolescent Psychiatric Disorders in Southeast Brazil. J. Am. Acad. Child Adolesc. Psychiatry.

[23] Cury C.R., Golfeto J.H. (2003). Strengths and difficulties questionnaire (SDQ): A study of school children in Ribeirão Preto. Rev. Bras. Psiquiatr..

[24] Szpalski M., Gunzburg R., Balagué F., Nordin M., Mélot C. (2002). A 2-year prospective longitudinal study on low back pain in primary school children. Eur. Spine J..

[25] Briggs A.M., Smith A.J., Straker L.M., Bragge P. (2009). Thoracic spine pain in the general population: Prevalence, incidence and associated factors in children, adolescents and adults. A systematic review. BMC Musculoskelet. Disord..

[26] Aartun E., Hartvigsen J., Wedderkopp N., Hestbaek L. (2014). Spinal pain in adolescents: Prevalence, incidence, and course: A school-based two-year prospective cohort study in 1300 Danes aged 11–13. BMC Musculoskelet. Disord..

[27] Savedra M.C., Tesler M.D., Ward J.D., Wegner C. (1988). How adolescents describe pain. J. Adolesc. Health Care.

[28] Goodman J.E., McGrath P. (1991). The epidemiology of pain in children and adolescents: A review. Pain.

[29] Meucci R.D., Linhares A.O., Olmedo D.W., Cousin S.E., Duarte V.M., Almeida C.J. (2018). Low back pain among adolescents in the semiarid region: Results of a population census in the city of Caracol, State of Piauí, Brazil. Cien. Saude. Colet..

[30] Torgén M., Swerup C. (2002). Individual factors and physical work load in relation to sensory thresholds in a middle-aged general population sample. Eur. J. Appl. Physiol..

[31] Ben Ayed H., Yaich S., Trigui M., Ben Hmida M., Ben Jemaa M., Ammar A., Jedidi J., Karray R., Feki H., Mejdoub Y. (2019). Prevalence, Risk Factors and Outcomes of Neck, Shoulders and Low-Back Pain in Secondary-School Children. J. Res. Health Sci..

[32] Trevelyan F.C., Legg S.J. (2011). Risk factors associated with back pain in New Zealand school children. Ergonomics.

[33] Viner R.M., Ozer E.M., Denny S., Marmot M., Resnick M., Fatusi A., Currie C. (2012). Adolescence and the social determinants of health. Lancet.

[34] Crombez G., Eccleston C., Van Damme S., Vlaeyen J.W., Karoly P. (2012). Fear-avoidance model of chronic pain: The next generation. Clin. J. Pain..

[35] Hartvigsen J., Hancock M.J., Kongsted A., Louw Q., Ferreira M.L., Genevay S., Hoy D., Karppinen J., Pransky G., Sieper J. (2018). What low back pain is and why we need to pay attention. Lancet.

[36] Lee H., Hubscher M., Moseley G.L., Kamper S.J., Traeger A.C., Mansell G., McAuley J.H. (2015). How does pain lead to disability? A systematic review and meta-analysis of mediation studies in people with back and neck pain. Pain.

[37] Edwards R.R., Dworkin R.H., Sullivan M.D., Turk D.C., Wasan A.D. (2016). The Role of Psychosocial Processes in the Development and Maintenance of Chronic Pain. J. Pain.

[38] Domoff S.E., Borgen A.L., Foley R.P., Maffett A. (2019). Excessive use of mobile devices and children’s physical health. Hum. Behav. Emerg. Technol..

[39] Berolo S., Wells R.P., Amick B.C. (2011). Musculoskeletal symptoms among mobile hand-held device users and their relationship to device use: A preliminary study in a Canadian university population. Appl. Ergon..

[40] Xie Y., Szeto G., Dai J. (2017). Prevalence and risk factors associated with musculoskeletal complaints among users of mobile handheld devices: A systematic review. Appl. Ergon..

[41] Eitivipart A.C., Viriyarojanakul S., Redhead L. (2018). Musculoskeletal disorder and pain associated with smartphone use: A systematic review of biomechanical evidence. Hong Kong Physiother. J..

[42] Roquelaure Y., Bodin J., Ha C., Le Marec F., Fouquet N., Ramond-Roquin A., Goldberg M., Descatha A., Petit A., Imbernon E. (2014). Incidence and Risk Factors for Thoracic Spine Pain in the Working Population: The French Pays de la Loire Study. Arthritis Care Res..

[43] Stallknecht S.E., Strandberg-Larsen K., Hestbæk L., Andersen A.-M.N. (2017). Spinal pain and co-occurrence with stress and general well-being among young adolescents: A study within the Danish National Birth Cohort. Eur. J. Pediatr..

[44] de Vitta A., Campos L.D., Bento T., Felippe L.A., Maciel N.M., Perrucini P. (2022). Thoracic Spine Pain and Factors Associated in High School Students. Pain Manag. Nurs..

[45] da Rosa B.N., Noll M., Candotti C.T., Loss J.F. (2022). Risk Factors for Back Pain among Southern Brazilian School Children: A 6-Year Prospective Cohort Study. Int. J. Environ. Res. Public Health.

[46] Miñana-Signes V., Monfort-Pañego M., Morant J., Noll M. (2021). Cross-Cultural Adaptation and Reliability of the Back Pain and Body Posture Evaluation Instrument (BackPEI) to the Spanish Adolescent Population. Int. J. Environ. Res. Public Health.

[47] Noll M., Candotti C.T., Rosa B.N.D., Vieira A., Loss J.F. (2021). Back pain and its risk factors in Brazilian adolescents: A longitudinal study. Br. J. Pain.

[48] Bento T.P.F., dos Santos Genebra C.V., Maciel N.M., Cornelio G.P., Simeão S.F.A.P., de Vitta A. (2020). Low back pain and some associated factors: Is there any difference between genders?. Braz. J. Phys. Ther..

[49] Santos E.D.S., Bernardes J.M., Noll M., Gómez-Salgado J., Ruiz-Frutos C., Dias A. (2021). Prevalence of Low Back Pain and Associated Risks in School-Age Children. Pain Manag. Nurs..

